# Supplementary material for: Heritability and complex segregation analysis of naturally-occurring diabetes in Australian Terrier Dogs
Source: PLoS One. 2020 Sep 24;15(9):e0239542. doi: 10.1371/journal.pone.0239542 (PMC7514011; doi:10.1371/journal.pone.0239542)
Supplement: S1 Appendix — (DOCX) [file pone.0239542.s001.docx]

Appendix 1

Academic veterinary hospitals and private veterinary referral hospitals, which promoted the online survey:

1. University of Alaska Veterinary Medicine Program (Dr. Arleigh Reynolds) – Fairbanks, AK 99775

2. Angell Animal Medical Center (Dr. Megan Whelan) – Boston, MA 02130

3. Animal Specialty Group (Dr. Rhonda Schulman) – Los Angeles, CA 90039

4. Auburn University College of Veterinary Medicine (Dr. Ellen Behrend) – Auburn, AL 36849

5. Blue Pearl Veterinary Partners (Brenda Fulcher) – Tampa, Florida 33614

6. University of California Davis School of Veterinary Medicine (Dr. Chen Gilor, Dr. Kate Hopper, Dr. Carrie Palm) – Davis, CA 95616

7. Charleston Veterinary Referral Center (Dr. Derek Duval) – Charleston, SC 29414

8. Colorado State University Veterinary Teaching Hospital (Dr. Craig Webb) – Fort Collins, CO 80525

9. Cornell University College of Veterinary Medicine (Dr. Daniel Fletcher) – Ithaca, NY 14853

10. Dogs and Cats Veterinary Referral (Dr. William Pullen) – Bowie, MD 20715

11. University of Florida College of Veterinary Medicine (Dr. Allison O’Kell, Dr. Richard Hill) – Gainesville, FL 32608

12. Four Seasons Veterinary Specialists (Dr. Lauren Prause) – Loveland, CO 80538

13. University of Georgia College of Veterinary Medicine (Dr. Cynthia Ward) – Athens, GA 30602

14. University of Illinois College of Veterinary Medicine (Dr. Heidi Phillips) – Urbana, IL 61802

15. IndyVet (Dr. Tracey Gillespie) – Indianapolis, IN 46203

16. Kansas State University College of Veterinary Medicine (Dr. Thomas Schermerhorn) – Manhattan, KS 66502

17. Louisiana State University College of Veterinary Medicine (Dr. Frederic Gaschen) – Baton Rouge, LA 70803

18. Metropolitan Veterinary Association (Dr. James Dougherty) – Norristown, PA 19403

19. Michigan State University Veterinary Medical Center (Dr. Matthew Beal) – East Lansing, MI 48824

20. Midwestern University (Dr. Angela Mexas) – Glendale, AZ 85308

21. University of Minnesota College of Veterinary Medicine (Dr. Eva Furrow & Dr. David Polzin) – St. Paul, MN 55108

22. Mississippi State University College of Veterinary Medicine (Dr. Patty Lathan) – Mississippi State, MS 39762

23. University of Missouri Veterinary Health Center (Dr. Leah Cohn) – Columbia, MO 65211

24. New England Animal Medical Center (Dr. Patricia Walters) – Bridgewater, MA 02379

25. North Carolina State University College of Veterinary Medicine (Dr. Shelly Vaden) – Raleigh, NC 27606

26. The Ohio State University Veterinary Medical Center (Dr. Cathy Langston) – Columbus, OH 43210

27. Oklahoma State University Boren Veterinary Medical Hospital (Dr. Mark Neer, Dr. Daniel Burba) – Stillwater, OK 74078

28. Oregon State University Carlson College of Veterinary Medicine (Dr. Helio de Morais, Dr. Jana Gordon) – Corvallis, OR 97331

29. University of Pennsylvania School of Veterinary Medicine (Dr. Rebecka Hess)

30. Purdue University College of Veterinary Medicine (Dr. Nolie Parnell) – West Lafayette, IN 47907

31. Seattle Veterinary Specialists (Dr. Frances Hurrell) – Seattle, WA 98104

32. Southeast Veterinary Oncology and Internal Medicine (Dr. Abigail Bertalan) – Orange Park, FL 32073

33. Specialized Veterinary Services (Dr. Melissa Tollett) – Fort Myers, FL 33912

34. University of Tennessee College of Veterinary Medicine (Dr. Jacqueline Whittemore) – Knoxville, TN 37996

35. Texas A&M College of Veterinary Medicine (Dr. Jorg Steiner) – College Station, TX 77843

36. Tufts Cummings School of Veterinary Medicine (Dr. Elizabeth Rozanski) – North Grafton, MA 01536

37. Tuskegee University Veterinary Medical Teaching Hospital (Dr. Jeannine Bellamy) – Tuskegee, AL 36088

38. Veterinary Specialists and Emergency Services (Dr. Michael Koch) – Brighton, NY 14623

39. Virginia-Maryland College of Veterinary Medicine (Dr. David Panciera) – Blacksburg, VA 24060

40. Washington State University College of Veterinary Medicine (Dr. Rance Sellon) – Pullman, WA 99164

41. Western University of Health Sciences: College of Veterinary Medicine (Dr. Linda Kidd) – Pomona, CA 91766

42. WestVet (Dr. Daniel Hume) – Garden City, ID 83714

43. University of Wisconsin School of Veterinary Medicine (Dr. Lauren Trepanier) – Madison, WI 53706
