# Supplementary material for: Heritability and complex segregation analysis of naturally-occurring diabetes in Australian Terrier Dogs
Source: PLoS One. 2020 Sep 24;15(9):e0239542. doi: 10.1371/journal.pone.0239542 (PMC7514011; doi:10.1371/journal.pone.0239542)
Supplement: Attachment — Submitted filename: Response to Reviewers.docx [file pone.0239542.s002.docx]

The authors thank the Editor and Reviewers for a concise, clear, insightful, and helpful review. All of the Reviewers’ comments have been addressed in the revised manuscript and are also listed below.

Reviewer 1:

1. They do not say whether this pedigree will be used to evaluate the identified genetic susceptibility region in the INS gene – which would be a valuable genetic analyses to perform for the Australian Terriers and may be of relevance to other diabetic susceptible breeds. While not part of this study, further genetic evaluation should be considered in the discussion.

The authors thank the reviewer for the suggestion. The following sentence has been added on lines 337-340 (in the revised manuscript with trach changes): “Sequencing of the 5.8 Mb insulin gene region in Australian Terriers and comparison of this sequence to that found in non-diabetic Australian Terriers holds particular promise for future genetic evaluations because this region has been associated with diabetes in this breed.”

Reviewer 2:

1. Lines 41-44- these sentences could be merged to aid flow

The authors thank the reviewer for the suggestion. The sentences on lines 41-43 (in the revised manuscript with trach changes) have been merged, as suggested, and now read as follows: “The Australian Terrier breed is the breed at highest risk for diabetes in Sweden, Australia, and the United States, where it is 32 times more likely to develop diabetes compared to mixed breed dogs [6, 7, 9].”

2. Lines 57-60 are a bit repetitive

Thank you for the suggestion. The following sentence (now on line 64) has been deleted, as suggested: “The heritability and polygenic mode of inheritance of diabetes in Australian Terriers are reported here.”

3. Im not sure that table 1 and 2 are adding much as they are just single line tables? These

maybe better in text, but I donʼt feel strongly and its up to you

Thank you for the suggestions. Table 1 has been deleted and replaced with the following sentence on lines 219-221: “Heritability of diabetes risk in Australian Terrier dogs in a mixed logistic regression model was estimated as 0.18 (95% posterior interval 0.00-0.67).”

Table 2 was also deleted and its contents were incorporated into the following sentence on lines 222-224: “There was no significant difference in the sex effect on the risk of diabetes detected in the mixed logistic regression model (0.37, 95% posterior interval -0.23-0.94).”

4. Line 284- does this genetic variability with diabetes also link to genetic variability of the

dogs? Is there inbreeding co-efficient known for these dogs?

Thank you for the query. The following sentence has been added to what is now lines 309-313, as suggested: “Making use of the available pedigree, all known relationships (including any inbreeding) were incorporated into the estimated heritability. Provided the pedigree in this study is a representative subset of all Australian Terriers, any impact of the presence of this inbreeding on our estimates would be expected to be negligible.”

5. Are there plans to use to genetic susceptibility data to prescreen animals for this disease? That would be a most useful disease diagnostic. Again- not something which needs commenting on, just a thought

The following sentence has been added to lines 313-315, as suggested: “The data generated here could also be used to calculate estimated breeding values of diabetes in US Australian Terriers, to help guide future breeding and decrease the risk of diabetes in this breed.”

6. It would be nice to see a bit more on further genetic evaluations which maybe useful in the

discussion too if you think it would be useful

The following sentence has been added on lines 337-340: “Sequencing of the 5.8 Mb insulin gene region in Australian Terriers and comparison of this sequence to that found in non-diabetic Australian Terriers holds particular promise for future genetic evaluations because this region has been associated with diabetes in this breed.”
